# Immunological impact of tumor-draining lymph node dissection on systemic Th1-like CD4^+^ T cells in patients with early-stage lung cancer

**DOI:** 10.1007/s00262-026-04357-4

**Published:** 2026-04-28

**Authors:** Atsushi Kamigaichi, Hiroshi Kagamu, Yoshihiro Miyata, Takahiro Mimae, Norifumi Tsubokawa, Koichi Hirano, Morihito Okada

**Affiliations:** 1 Department of Surgical Oncology, Hiroshima University, 1-2-3 Kasumi, Minami-ku, Hiroshima, 734-8551 Japan; 2 Division of Respiratory Medicine, Saitama Medical University International Medical Center, Saitama, Japan

**Keywords:** Tumor-draining lymph node, Early-stage lung cancer, Antitumor immunity, CD4^+^ T cell

## Abstract

**Introduction:**

Tumor-draining lymph nodes (LNs) are critical for initiating and maintaining antitumor immunity. However, systematic LN dissection (LND) remains the standard surgical procedure for lung cancer. This study aimed to investigate the immunological impact of tumor-draining LND on systemic T cell subsets.

**Methods:**

We prospectively analyzed perioperative peripheral blood and resected LN samples from patients with early-stage lung adenocarcinoma who underwent lobectomy with systematic LND (systematic LND group) or wedge resection without LND (LN-preserving group). Perioperative immune cell dynamics were comprehensively profiled using mass cytometry.

**Results:**

Unlike conventional CD4^+^ and CD8^+^ T cell subsets, Th7R (CXCR3^±^CCR4^−^CCR6^+^ CD62L^low^CD4^+^ T cell), a Th1-like CD4^+^ T cell cluster essential for antitumor immunity, consistently decreased in peripheral blood after tumor resection in both groups (*p* = 0.0016 and *p* = 0.0033). This decline was significantly milder in the LN-preserving group than in the systematic LND group (*p *= 0.0153). In LN analyses, Th7R percentages were significantly higher in hilar, interlobar, and peripheral LNs than in subcarinal and other mediastinal LNs (*p *= 0.0148). Th7R percentages in resected LNs strongly and negatively correlated with postoperative changes in peripheral blood (*r *= −0.857, *p* = 0.0137). Furthermore, greater declines in Th7R in peripheral blood were associated with postoperative oncological disease events.

**Conclusions:**

Preserving LNs contributes to maintaining systemic antitumor immunity after surgery for early-stage lung cancer. Hilar, interlobar, and peripheral LNs may serve as primary reservoirs and supply sources of Th7R. In early-stage disease, these findings suggest a potential immunological benefit of LN-preserving strategies.

**Supplementary Information:**

The online version contains supplementary material available at 10.1007/s00262-026-04357-4.

## Introduction

The cancer–immunity cycle is an interactive process wherein T cell-mediated tumor cell destruction involves subsequent rounds of antigen presentation and lymphocyte stimulation, sustaining an adaptive immune attack against the tumor [1]. This cycle critically depends on a coordinated T cell response, in which cytotoxic CD8^+^ T cells directly eliminate malignant cells, whereas CD4^+^ T cells play an indispensable role in shaping the response, including licensing dendritic cells to effectively prime CD8^+^ T cells [2, 3]. Consequently, systemic CD4^+^ T cell immunity correlates with tumor eradication and survival outcomes following immunotherapy, underscoring its importance in successful cancer immunotherapy [4–6]. Recent studies have highlighted the critical role of Th type 1 (Th1)-like CD4^+^ T cells in antitumor immunity [6–8]. Single-cell RNA sequencing and mass cytometry of peripheral blood from patients with advanced lung cancer receiving PD-1 blockade revealed a distinct T-bet-positive Th1-like cluster [6]. This cluster is epigenetically and transcriptionally unique compared to canonical Th1 cells and possesses a different T cell receptor clonotype. Trajectory analysis identified a single functional meta-cluster situated between Th1 and Th17 expression profiles, composed of CXCR3^+^CCR4^−^CCR6^+^ cells (Th1/17) and CXCR3^−^CCR4^−^CCR6^+^ cells (CCR6 SP). This cluster was named Th7R because of high IL-7 receptor expression [6]. Pretreatment presence of Th7R, but not Th1, in peripheral blood is correlated with therapeutic efficacy following PD-1 blockade therapy [6].

Although the tumor microenvironment (TME) is a primary site of immune interaction, tumor-draining lymph nodes (TDLNs) are increasingly recognized as essential hubs for the antitumor immune response [9]. TDLNs function as critical sites for priming and maintaining tumor-specific T cell populations that serve as reservoirs sustaining ongoing immune attacks [10, 11]. Preclinical evidence indicates that PD-1/PD-L1 blockade efficacy depends on TDLN presence, as their surgical resection can abolish therapy-induced tumor regression [12, 13]. Despite this crucial immunological function, systematic lymph node dissection (LND) remains the standard surgery for early-stage non-small cell lung cancer to ensure accurate pathological staging [14]. Although previous reports show reduced effector T cells after lung cancer surgery [7, 15], the immunological impact of TDLN dissection on systemic antitumor immunity, including critical effector T cell populations, remains poorly understood.

Therefore, this study aimed to elucidate the immunological impact of TDLN dissection by comprehensively profiling perioperative systemic T cell subsets using mass cytometry, with a focus on CD4^+^ T cells essential for inducing and sustaining CD8^+^ T cell immunity.

## Materials and methods

### Study design and patient cohort

**Fig. 1:**
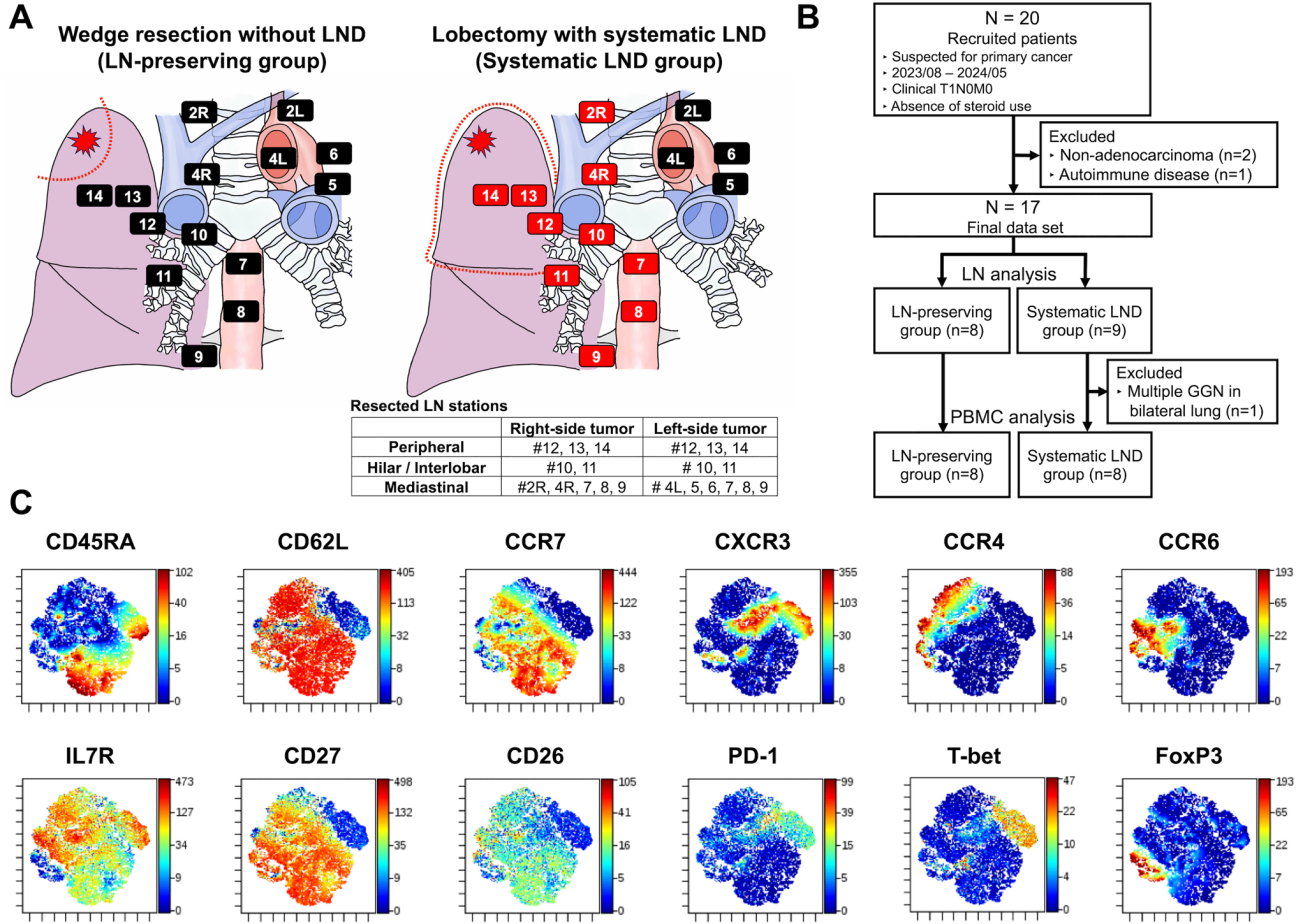
Study population and CD4^+^ T cell clusters **A**, Extent of lymph node dissection (LND) in the LN-preserving and systematic LND groups. **B**, CONSORT flow diagram illustrating the recruitment of eligible patients, reasons for exclusion, and the final patient cohorts for LN analysis (*n* = 17) and peripheral blood mononuclear cell (PBMC) analysis (*n* = 16). **C**, Representative viSNE figures of gated CD4^+^CD3^+^ cells from PBMC

This study adhered to the amended Declaration of Helsinki guidelines and was approved by the Institutional Review Board of Hiroshima University Hospital (approval date: May 30, 2023; approval code: E-2022–0278). All participants provided written informed consent prior to enrollment. The study included patients aged > 18 years with suspected clinical stage IA lung cancer and no suspicion of lymph node (LN) metastasis who were scheduled for lobectomy with systematic LND (systematic LND group) or wedge resection without LND (LN-preserving group) (Fig. 1A). Eligible patients had tumors with a ground-glass opacity component, indicating lung adenocarcinoma at low risk of LN metastasis [16]. Patients with hematological disorders, concurrent malignancies, or those receiving steroid therapy were excluded from the study.

### Sample collection

Peripheral blood mononuclear cell (PBMC) samples were collected from each patient at two time points—within 1 day before surgery and 1 week after surgery—using heparinized CPTV vacutainer tubes (Becton Dickinson Vacutainer Systems), as described previously [5, 7]. Samples were frozen using Cell Banker 2 (Nippon.

Zenyaku Kogyo Co.) in liquid nitrogen. For the T cell subset analysis, cells were incubated for 32–48 h in RPMI 1640 medium supplemented with 10% FCS before staining.

In the systematic LND group, LNs were surgically resected for standard clinical care, and lymphocytes were isolated from LN tissue. Single-cell suspensions were prepared mechanically by teasing the collected LNs with needles and pressing tissue fragments using the blunt end of a 10-mL plastic syringe.

### Extent of LND

In the systematic LND group, all patients underwent lobectomy involving dissection of the hilar and mediastinal LNs according to the International LN map (Fig. 1A) [17, 18]. During resection of right-sided tumors, upper mediastinal LNs, including the right upper and lower paratracheal nodes (stations #2R, 4R), subcarinal (station #7), and lower mediastinal LNs (stations #8, 9), were dissected. During resection of the left-sided tumors, upper mediastinal LNs, including the left upper paratracheal node (station # 4L), aortic nodes in the anteroposterior zone (stations #5 and 6), subcarinal (station #7), and lower mediastinal LNs (stations #8, 9) were dissected. The upper, lower mediastinal, and subcarinal LNs were classified as N2 nodes. N1 nodes, including hilar (station #10), interlobar zone (station #11), and peripheral zone (stations #12–14) nodes, were routinely resected on both sides [17–19]. The LNs were resected en bloc with surrounding fatty tissue.

### Mass cytometry analysis

Mass cytometry analysis was performed using the Helios system (Standard BioTools) as previously described [5–7]. Following thawing of cryopreserved PBMCs, up to 5.0 × 10^6^ cells were stained for viability with ^198^Pt monoisotopic cisplatin before incubation with a metal-conjugated antibody cocktail for surface marker detection. For intracellular targets such as T-bet and FOXP3, cells were fixed and permeabilized using the Maxpar Nuclear Antigen Staining Buffer kit. The final fixation step used Maxpar Fix and Perm Buffer containing 125 nM iridium nucleic acid intercalator. After a series of washes, cells were resuspended in Maxpar water for data acquisition. Collected cells from each sample were analyzed using the Helios and Cytobank software for high-dimensional visualization with viSNE based on t-distributed stochastic neighbor embedding algorithm. The gating strategy was applied as previously described [5–7].

### Statistical analysis

This study involved PBMC analysis comparing perioperative changes in peripheral blood immune phenotypes between the systematic LND and LN-preserving groups, LN analysis comparing immune phenotypes across LN stations, and correlation analysis assessing the correlation between immune phenotypes in the peripheral blood and LNs. Data are expressed as the mean (± SEM), unless indicated otherwise. A paired *t* test was used to compare the differences in immune phenotypes between preoperative and postoperative blood samples. Analysis of covariance (ANCOVA) was used to compare postoperative changes between the groups while adjusting for baseline values. Welch’s t test was used to compare immune phenotypes between LNs. Correlation analysis was performed using Spearman’s rank correlation coefficient. Survival curves were estimated using the Kaplan–Meier method. Statistical analyses were performed using JMP version 16 (SAS Institute, Cary, NC, USA). All figures were generated using Prism 9 software (GraphPad Software, Inc., La Jolla, CA, USA). Statistical significance was set at *p* < 0.05.

## Results

### Patient characteristics

Twenty patients meeting the eligibility criteria were enrolled between August 2023 and May 2024. After excluding two patients with non-adenocarcinoma histology and one with concurrent autoimmune disease, the final dataset for LN analysis included 17 patients. For PBMC analysis, one patient with multiple ground-glass nodules in the residual lung was further excluded due to persistent cancer antigens, resulting in a cohort of 16 patients, with 8 each in the systematic LND and LN-preserving groups (Fig. 1B).

The baseline characteristics of the 16 patients included in the PBMC analysis are summarized in Table 1. No significant differences were observed in age, sex, complication, or pathological stage between the two surgical groups. However, the systematic LND group exhibited significantly longer operative time, greater blood loss, and larger tumor volume than the LN-preserving group.

**Table 1:** Patient characteristics in the analysis set of peripheral blood

	Wedge resection without LND (LN-preserving group) (*n* = 8)	Lobectomy with systematic LND (Systematic LND group) (*n* = 8)	*p* - value
Age, years	77.5 [68.3–82.0]	72 [64.3–77.0]	0.155
*Sex*			
Male	6 (75.0)	4 (50.0)	0.608
*Smoking history*			
Ever	4 (50.0)	5 (62.5)	1.0
*Tumor location*			
Upper / Middle / Lower lobe	5 (62.5) / 0 / 3 (37.5)	3 (37.5) / 3 (37.5) / 2 (25.0)	0.282
Consolidation-to-tumor ratio	0.49 [0.15–0.72]	0.74 [0.47–0.91]	0.115
Surgical time, min	57 [53–79]	138 [104–184]	0.006
Bleeding, mL	10 [5–24]	26 [21–106]	0.004
Postoperative complication	1 (12.5)	1 (12.5)	1.0
*Pathological stage*			
0 / I	2 (25.0) / 6 (75.0)	1 (12.5) / 7 (87.5)	0.519
Tumor volume (cm^3^)	1.21 [0.63–1.99]	2.84 [2.56–5.34]	0.014
*PD-L1 expression level*			0.308
< 1% / 1–49% / > 50%	6 (75.0) / 1 (12.5) / 0	2 (25.0) / 2 (25.0) / 1 (12.5)	
Unknown	1 (12.5)	3 (37.5)	
*EGFR mutation*			1.0
Present / Absent	1 (12.5) / 6 (75.0)	2 (25.0) / 5 (62.5)	
Unknown	1 (12.5)	1 (12.5)	

EGFR, epidermal growth factor receptor

^*^Categorical data are shown as numbers (%) and continuous data as medians (interquartile ranges)

### Characterization of the CD4^+^ and CD8^+^ T cell subsets in peripheral blood and LNs

To comprehensively characterize the T cell landscape, T cells in the peripheral blood were analyzed using mass cytometry. The results revealed distinct cell clusters through viSNE analysis of CD4^+^ and CD8^+^ T cells (Fig. 1C; Supplementary Figure S1). Within the CD62L^low^CD4^+^ T cell subsets, we identified a specific cluster phenotypically consistent with Th7R subsets (CXCR3^±^CCR4^−^CCR6^+^ CD62L^low^CD4^+^ T cells), which play a promising role in antitumor immunity [6]. Heatmap analysis of protein expression confirmed that CCR6 SP and Th1/17, comprising the Th7R cells, maintained a consistent effector phenotype characterized by high expression of the IL-7 receptor (CD127) and CXCR4 across preoperative peripheral blood, hilar/interlobar (N1) LNs, and mediastinal (N2) LNs, compared with conventional Th1 or Th17 subsets (Supplementary Figure S2).

### Postoperative changes of T cell subsets in peripheral blood

**Fig. 2:**
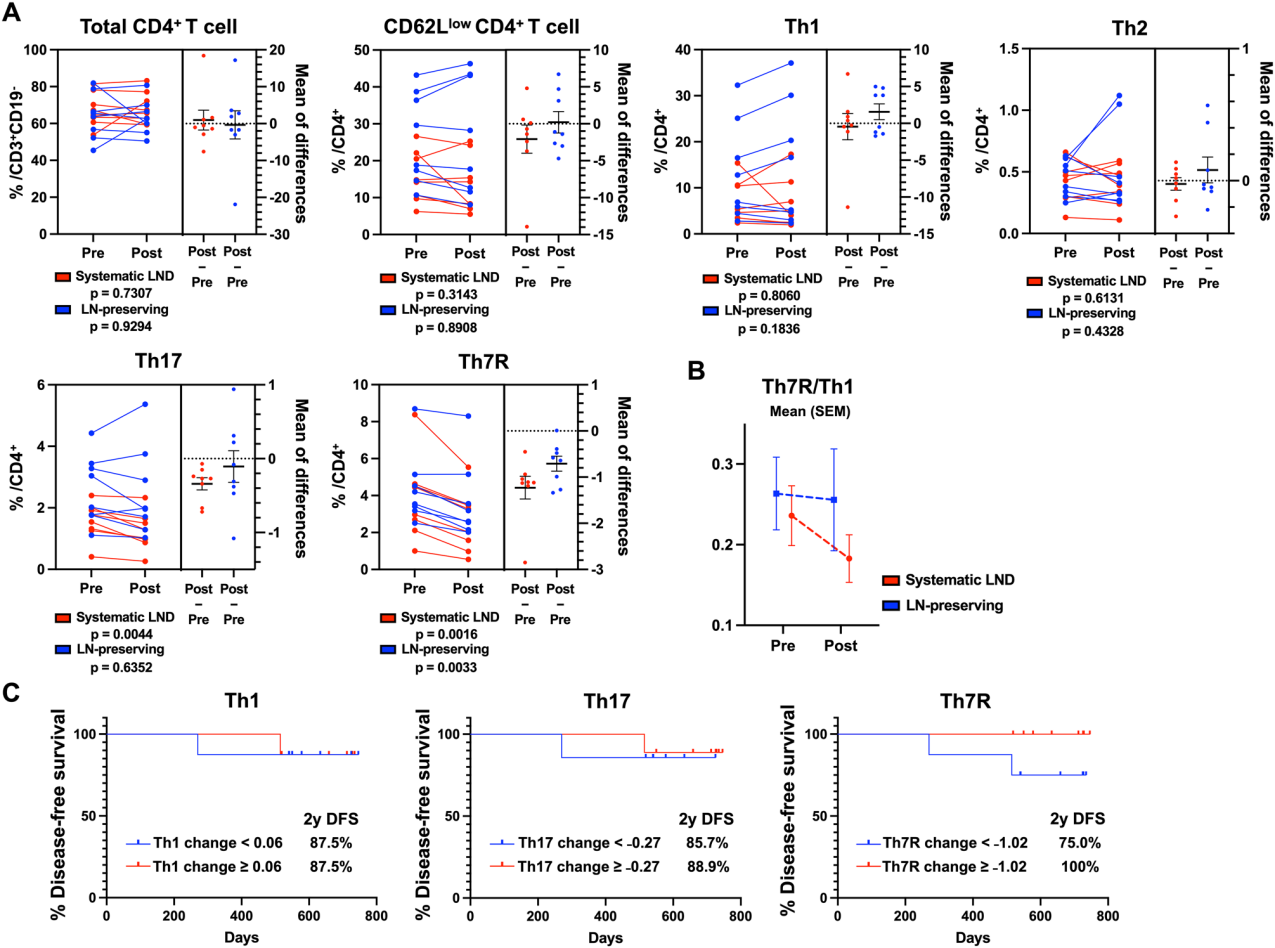
Postoperative dynamics of CD4^+^ T cell subsets in peripheral blood after lung cancer surgery **A**, Paired plots showing the preoperative and postoperative percentages of total CD4^+^ T cell, CD62L^low^CD4^+^ T cell, Th1, Th2, Th17, and Th7R in systematic lymph node dissection (LND) group (red lines) or LN-preserving group (blue lines). The right panels show the individual postoperative changes, calculated as the postoperative minus preoperative percentages. **B**, Paired analysis of the Th7R/Th1 ratio before and after surgery. Data are presented as mean (SEM). *P* values for paired comparisons were determined using the paired t test. **C,** Kaplan–Meier curves for disease-free survival (DFS) comparing patients stratified by postoperative changes in Th1, Th17, and Th7R in peripheral blood. The cutoff value was based on the median value of postoperative change (postoperative minus preoperative: Th1, 0.06%; Th17, − 0.27%; Th7R, − 1.02%). Patients were classified into the decreased (change < median) and maintained (change ≥ median) groups for each subset

We then evaluated the impact of LND on systemic T cell subsets in peripheral blood. The percentages of total CD4^+^ T cells, Th1 (CXCR3^+^CCR4^−^CCR6^−^ CD62L^low^CD4^+^ T cells), Th2 (CXCR3^−^CCR4^+^CCR6^−^ CD62L^low^CD4^+^ T cells), and Th17 (CXCR3^−^CCR4^+^CCR6^+^ CD62L^low^CD4^+^ T cells) subsets showed considerable interpatient variability and no consistent postoperative change (Fig. 2A), although Th17 significantly decreased in the systematic LND group (*p* = 0.0044). Similarly, no significant postoperative changes were observed in the percentages of total CD8^+^ T cells or their subsets, including central memory (CM: CCR7^+^CD45RA^−^), effector memory (EM: CCR7^−^CD45RA^−^), and effector memory re-expressing CD45RA (EMRA: CCR7^−^CD45RA^+^) CD8^+^ T cells (Supplementary Figure S3). Conversely, the Th7R subset exhibited a uniform trend. Specifically, the percentage of Th7R significantly decreased postoperatively in both the systematic LND (*p* = 0.0016) and LN-preserving groups (*p* = 0.0033). However, baseline-adjusted comparison of the magnitude of decline between groups using ANCOVA showed a significantly milder decrease in systemic Th7R levels in the LN-preserving group (between-group difference, 0.58; 95% confidence interval, 0.13–1.02; *p* = 0.0153) (Fig. 2A; Table 2), suggesting a protective effect of LN preservation on antitumor immunity.

**Table 2:** Postoperative change of CD4^+^ T cell subpopulation in peripheral blood

	Preoperative, %	Postoperative, %	Pre vs. post	Post−pre, %		Group effect
	Mean (SEM)	Mean (SEM)	(*p* - value^*^)	Mean (95% CI)	Between-group diff. (95% CI)^†^	(*p* - value^‡^)
*CD4*^+^ *T cell*						
Systematic LND	67.5 (9.1)	68.4 (8.5)	0.7307	0.96 (− 5.39–7.32)	− 3.13 (− 11.78–5.53)	Baseline effect: 0.0295
LN-preserving	63.9 (12.5)	63.5 (9.3)	0.9294	− 0.35 (− 9.36–8.66)		Group effect: 0.4467
*CD62L*^*low*^* CD4*^+^ *T cell*						
Systematic LND	15.6 (6.9)	13.5 (7.7)	0.3143	− 2.07 (− 6.59–2.45)	1.75 (− 3.92–7.43)	Baseline effect: < 0.0001
LN-preserving	26.2 (12.4)	26.4 (16.0)	0.8908	0.21 (− 3.22–3.63)		Group effect: 0.5131
*Th1*						
Systematic LND	6.9 (4.7)	6.4 (5.4)	0.8060	− 0.45 (− 4.66–3.75)	2.58 (− 2.06–7.22)	Baseline effect: 0.0001
LN-preserving	13.3 (10.7)	14.9 (13.4)	0.1836	1.57 (− 0.95–4.10)		Group effect: 0.2487
*Th2*						
Systematic LND	0.4 (0.2)	0.4 (0.2)	0.6131	− 0.03 (− 0.14–0.09)	0.10 (− 0.14–0.34)	Baseline effect: 0.0101
LN-preserving	0.4 (0.2)	0.5 (0.4)	0.4328	0.08 (− 0.15–0.31)		Group effect: 0.3649
*Th17*						
Systematic LND	1.6 (0.6)	1.2 (0.6)	0.0044	− 0.34 (− 0.54 to − 0.15)	0.16 (− 0.47–0.79)	Baseline effect: < 0.0001
LN-preserving	2.6 (1.1)	2.5 (1.5)	0.6352	− 0.11 (− 0.61–0.40)		Group effect: 0.5888
*Th7R*						
Systematic LND	3.9 (2.2)	2.6 (1.6)	0.0016	− 1.23 (− 1.81 to − 0.65)	0.58 (0.13–1.02)	Baseline effect: < 0.0001
LN-preserving	4.4 (1.9)	3.7 (2.1)	0.0033	− 0.71 (− 1.09 to − 0.33)		Group effect: 0.0153

Th1, CXCR3^+^CCR4^−^CCR6^−^ CD62L^low^CD4^+^ T cell; Th2, CXCR3^−^CCR4^+^CCR6^−^ CD62L^low^CD4^+^ T cell; Th17, CXCR3^−^CCR4^+^CCR6^+^ CD62L^low^CD4^+^ T cell; Th7R, CXCR3^±^CCR4^−^CCR6^+^ CD62L^low^CD4^+^ T cell; CI, confidence interval; Diff, difference; LN, lymph node; LND, lymph node dissection

^*^p values were calculated using the paired t test for pre- versus. postoperative values within each group

^†^Between-group differences were calculated using analysis of covariance, with preoperative values used as a covariate

^‡^*p* values were calculated using analysis of covariance, with preoperative values used as a covariate

Furthermore, Th7R is important for TME formation and may provide long-lasting antitumor immune responses in advanced lung cancer [5, 6]. Given that Th1 cells mediate immediate antitumor effects, the Th7R/Th1 ratio may reflect the balance between long- and short-term effector functions in antitumor immunity. The Th7R/Th1 ratio significantly decreased after systematic LND (*p* = 0.0099) but was maintained in the LN-preserving group (*p* = 0.8411) (Fig. 2B). Therefore, LN preservation may sustain a balance toward long-term antitumor immunity.

### Association of postoperative change of CD4^+^ and CD8^+^ T cell subsets with clinical outcome

We assessed the prognostic relevance of postoperative dynamics of CD4^+^ and CD8^+^ T cell subsets in the peripheral blood. Patients were stratified into decreased and maintained groups based on the median value of postoperative change for each subset (Th1: 0.06%; Th17: − 0.27%; Th7R: − 1.02%; CM: − 1.14%; EM: 1.20%; EMRA: − 0.45%). During the follow-up period (median, 686 days), two events (one recurrence and one second primary lung cancer) occurred in the Th7R decreased group (2-year disease-free survival [DFS] rate: 75.0%), whereas no events occurred in the Th7R maintained group (2-year DFS rate: 100%) (Fig. 2C). Conversely, postoperative dynamics of Th1 and Th17 in the CD4^+^ T cell subsets were not associated with clinical outcomes. The 2-year DFS rates were comparable between the decreased and maintained groups for Th1 (87.5% Vs. 87.5%) and Th17 cells (85.7% Vs. 88.9%) (Fig. 2C). Similarly, among CD8^+^ T cell subsets, 2-year DFS rates were comparable between decreased and maintained groups for CM, EM, and EMRA (all 87.5%) (Supplementary Figure S4).

### Immunological phenotype in LNs

**Fig. 3:**
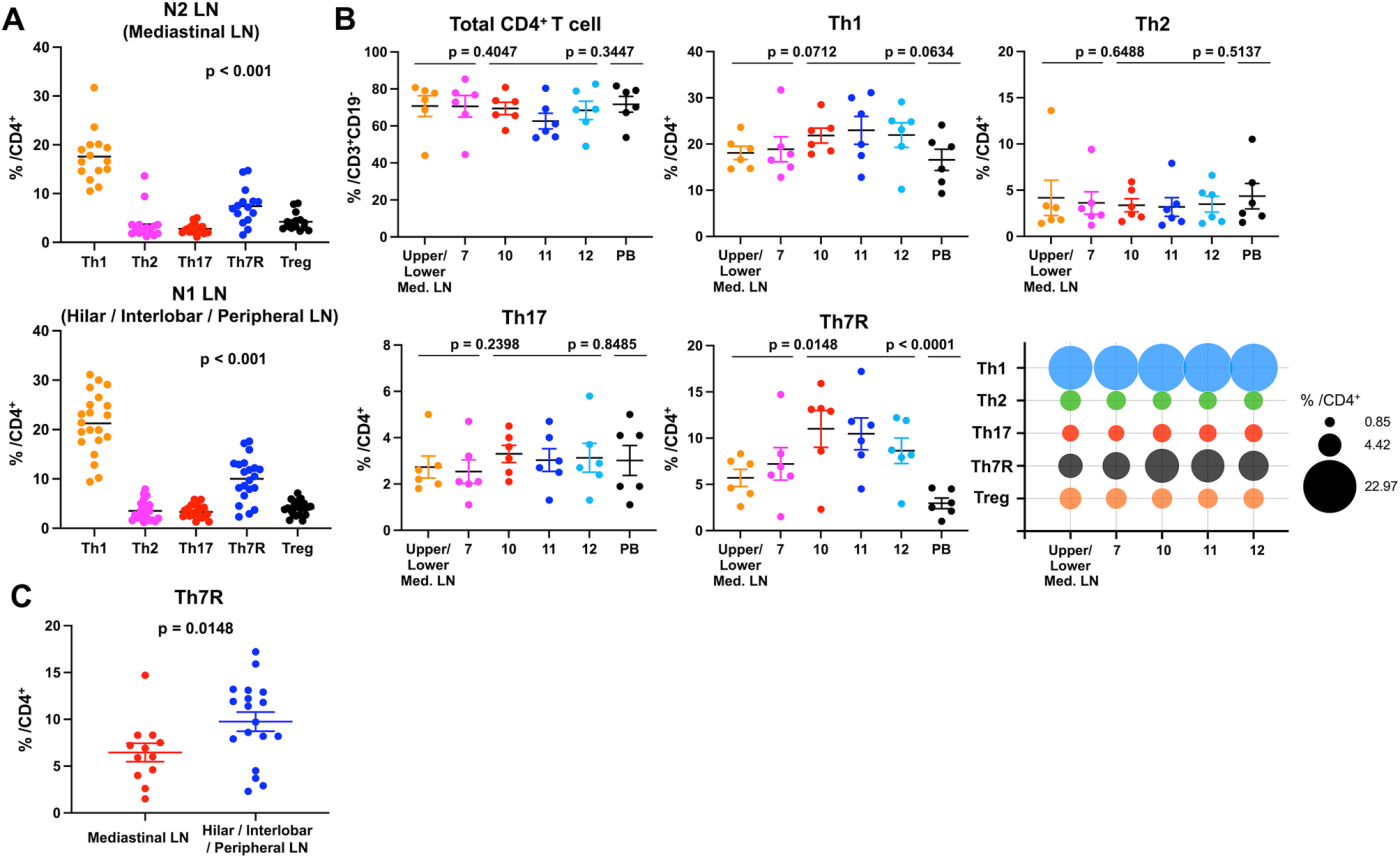
Immunological phenotype in lymph nodes (LNs) **A**, Dot plots illustrating the percentages of major CD4^+^ T cell subsets in N2 (mediastinal) and N1 (hilar/interlobar/peripheral) LNs. p values indicate comparison between Th7R versus Th2 and Th17. **B,** Differences in immunological phenotypes across individual LNs and peripheral blood (PB). Bubble plot illustrating the relative abundance and distribution of T cell subsets across LN stations. The size of the bubble corresponds to the mean percentage. **C**, Dot plots illustrating the percentage of Th7R (among total CD4^+^ T cells) in N2 (mediastinal) and N1 (hilar/interlobar/peripheral) LNs. p values were calculated using the Welch t test

To investigate the role of intrathoracic LNs, the composition of the CD4^+^ T cell subsets within each LN station resected in the systematic LND group was analyzed. In both N1 and N2 LNs, Th1 was the most common CD4^+^ T cell subset, whereas Th7R was more abundant than Th2, Th17, and Treg cells (Fig. 3A). Detailed analysis of subdivided LN stations revealed that percentages of total CD4^+^ T cells, Th1, Th2, and Th17 did not differ significantly across LN stations and peripheral blood (Fig. 3B). However, Th7R percentages were significantly higher in hilar (station #10), interlobar (station #11), and peripheral (station #12) LNs than in mediastinal LNs (subcarinal [station #7] and other mediastinal LNs) (p = 0.0148) and peripheral blood (p < 0.0001) (Fig. 3B, C). Notably, Th7R levels were comparable across N1 stations, with no significant differences between hilar (station #10) and interlobar (station #11) LNs (p = 0.624) or between interlobar (station #11) and station #12 LNs (p = 0.724). These findings indicate that hilar, interlobar, and peripheral LNs belonging to N1 LNs function as storage and supply sites for Th7R as TDLNs.

### Correlation of CD4^+^ T cell subsets between peripheral blood and LN

**Fig. 4:**
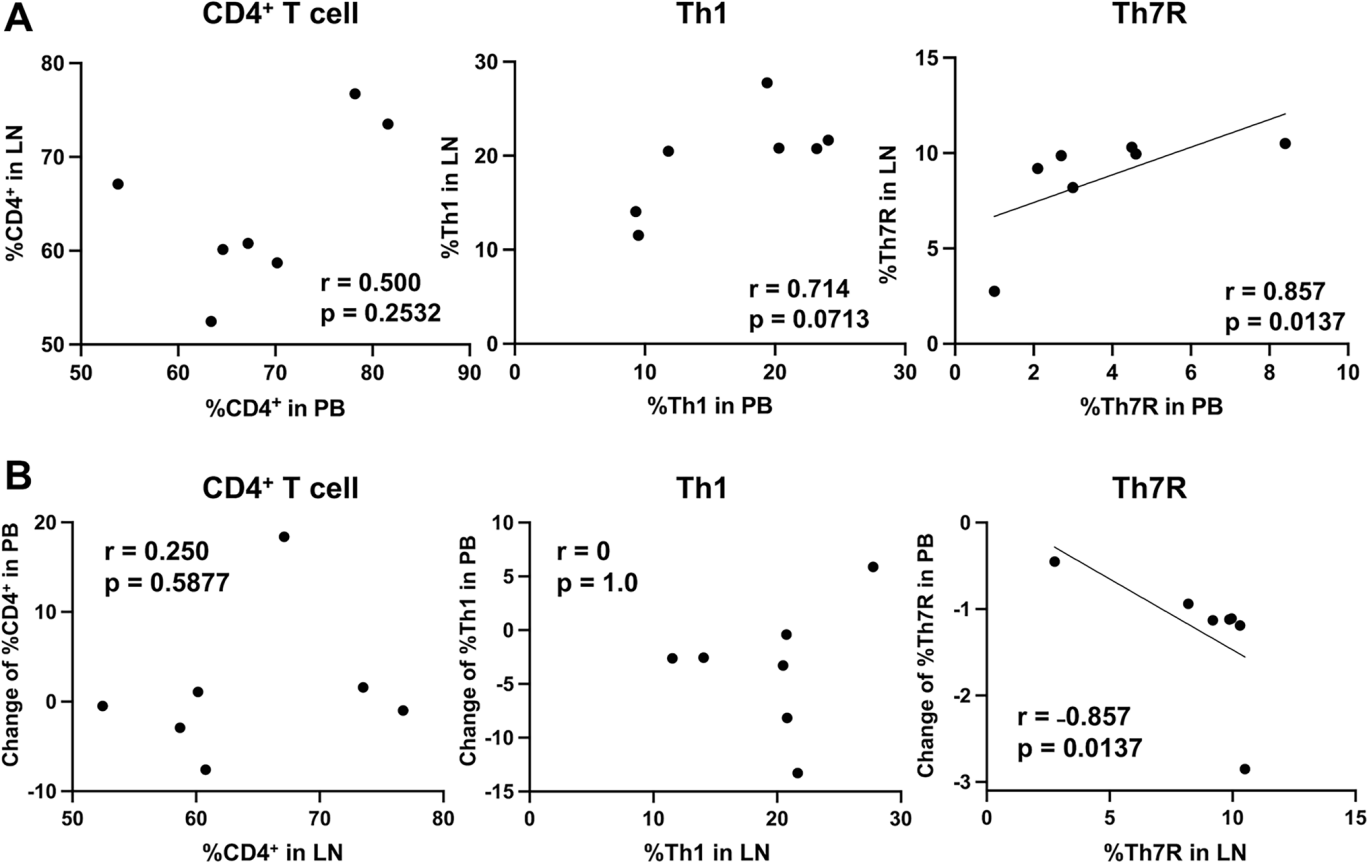
Correlation between the percentages of CD4^+^ T cells, Th1, and Th7R in lymph nodes (LNs) and peripheral blood (PB) **A**, Scatter plot showing the correlation between the percentage of CD4^+^ T cell subsets in resected LNs and their corresponding percentage in preoperative PB. **B**, Scatter plot showing the correlation between percentages of CD4^+^ T cell subsets in resected LNs and corresponding postoperative changes (%) in peripheral blood. The Spearman correlation coefficient (r) and p value are shown for each plot

A correlation analysis was performed in seven patients with fully paired peripheral blood and LN samples. The percentages of total CD4^+^ T cells and Th1 cells in preoperative peripheral blood showed positive correlations with resected LNs (*r* = 0.500, *p* = 0.2532, and *r *= 0.714, *p* = 0.0713, respectively). Specifically, a strong positive correlation was observed for Th7R between preoperative peripheral blood and resected LNs (*r* = 0.857, *p* = 0.0137) (Fig. 4A). A very strong negative correlation was observed between postoperative changes in peripheral blood Th7R and their percentages within resected LNs (*r* = − 0.857, *p* = 0.0137) (Fig. 4B). This indicates that greater Th7R removal via LN dissection results in a larger subsequent decline in peripheral blood. No correlation was observed for total CD4^+^ T cells (*r* = 0.250, *p* = 0.5877) or Th1 cells (*r *= 0, *p* = 1.0). Additionally, no significant correlation was observed between consolidation-to-tumor ratio, tumor volume, operative time, blood loss, and postoperative Th7R changes in peripheral blood (Supplementary Figure S5), suggesting that observed differences in Th7R dynamics between the groups were not primarily driven by tumor size or surgical invasiveness.

## Discussion

This study provides clinical insights into the impact of TDLN dissection on systemic antitumor immunity in patients undergoing surgery for early-stage lung cancer. First, unlike conventional CD8^+^ T cell and Th1, Th2, or Th17 CD4^+^ T cell subsets, the systemic population of Th7R (Th1-like CD4^+^ T cell clusters) in peripheral blood decreased postoperatively regardless of LN removal, consistent with a previous report [7]. However, this decline was significantly milder in the LN-preserving group than in the systematic LND group. Additionally, Th7R percentages in TDLNs were strongly correlated with those in peripheral blood. Hence, preservation of tumor-draining LNs may help maintain host systemic antitumor immunity. Furthermore, our findings provide an immunological LN map indicating that hilar, interlobar, and peripheral LNs may function as primary TDLNs in early-stage lung cancer.

These findings are supported by the presence of a unique Th1-like CD4^+^ T cell population, defined as Th7R, which plays a role in antitumor immunity. Th7R is a functional meta-cluster distinct from classical Th1 and Th17 lineages in epigenetic profile and T cell receptor clonotype [6]. A defining molecular signature of Th7R is high co-expression of the IL-7 receptor (IL7R) and transcription factor TCF7 [6]. These molecules are crucial for conferring a durable, self-renewing phenotype. IL7R and TCF7 promote survival and proliferation of antitumor T cells, and IL7R signaling is essential for the efficacy of immune checkpoint inhibitors (ICIs) [20–22]. This capacity for persistence is further supported by the observation that, upon stimulation, Th7R upregulates survival-associated genes, such as *MYC*, *BATF*, and *BIRC3* [6]. Furthermore, Th7R shapes immune responses within the TME through CXCL13 and lymphotoxin-β expression [7]. These molecules are essential for forming high endothelial venules and tertiary lymphoid structures (TLSs), which recruit key antitumor lymphocytes, including PD-1^+^TCF-1^+^ precursor exhausted CD8^+^ T cells (Tpex), into tumors [23–25]. Tpex selectively localizes to TLS within the TME [26]. Given its characteristics, Th7R may also reside within TLS alongside Tpex, where it is maintained and amplified. The elucidation of Th7R dynamics provides key insights into the dynamic changes in specific antitumor immunity.

TDLNs are essential for priming and reinvigorating T cells and for successful ICI therapy [9, 12]. Tumor-specific CD8^+^ T cells in TDLNs are clonally related to those within tumors [11]. TDLNs function as reservoirs for stem-like T cell precursors that sustain immune responses by continuously supplying effector cells to tumors and systemic circulation [10, 11]. Based on the hypothesis that Th7R, like Tpex cells, is maintained and amplified in TLS, surgical tumor resection inherently entails TLS loss, which may lead to systemic decline in Th7R. However, our finding that TDLN preservation significantly mitigated this postoperative systemic decline provides insight into TDLN function, not merely as sites of cancer metastasis but also as sites for maintaining systemic antitumor immunity. This specificity is underscored by the finding that other systemic T cell populations, including total CD4^+^ T cells, Th1, Th2, Th17, and conventional CD8^+^ T cell subsets, showed no consistent postoperative changes following LND. Furthermore, unlike total CD4^+^ T cell and Th1 cells, Th7R percentages in resected LNs were strongly correlated with postoperative declines in peripheral blood, reaffirming that TDLNs serve as a key reservoir and supply source for tumor-specific T cell subsets.

A previous study identified immunological differences between station #12 and ipsilateral mediastinal LNs [7]. However, detailed and subdivided immunological status for each LN station based on the LN map is lacking. This station-specific information is essential for reconsidering surgical strategies, particularly for the LND extent. In our study, Th7R was significantly more concentrated in hilar, interlobar, and peripheral N1 LNs (stations #10, #11, and #12) than in mediastinal N2 LNs (station #7 and others), suggesting that these LNs may function as primary TDLNs. These findings have significant implications for the clinical management of early-stage lung cancer. Within the context of early-stage disease evaluated in this cohort, our study provides a specific and clinically relevant rationale for re-evaluating the value of LND while balancing oncological and immunological perspectives. Furthermore, the importance of TDLN preservation is increasingly recognized in the broader context of the perioperative immunotherapy era [27–30]. This is supported by preclinical studies demonstrating diminished ICI efficacy following lymph node resection [12, 13], as well as clinical data suggesting that neoadjuvant ICI administration (before TDLN removal) is superior to the adjuvant setting [31]. While our results do not directly support practice-changing surgical modifications in advanced stages, future research is expected to investigate the potential immunological benefits of LN-preserving strategies, including in locally advanced lung cancer.

The strength of this study lies in prospective patient selection focusing on patients with N0 early-stage lung adenocarcinomas. The presence of metastatic LNs results in the loss of antitumor immune function, fostering a negative immune microenvironment [11, 32]. Therefore, investigation of patients free of lymphatic metastasis is necessary to accurately assess the impact of functional TDLNs on systemic immune status. Nevertheless, this study has some limitations. First, the sample size was relatively small, making our results primarily exploratory. Particularly, the survival analysis in this study is preliminary, as the extremely low number of events precluded adjustments for potential confounders. Second, inherent selection bias exists due to the non-randomized nature of this study. Surgical procedures were determined based on clinical indications; consequently, the systematic LND group had significantly higher consolidation-to-tumor ratios and larger tumor volumes and underwent more invasive surgery, characterized by longer operative times and increased blood loss, compared with the LN-preserving group. Although our correlation analyses suggested that Th7R dynamics were not primarily driven by these factors, the potential influence of differences in the detailed TME or surgical stress cannot be completely excluded. Third, whether these findings can be extrapolated to other histological types, such as squamous cell carcinoma or node-positive disease, remains unclear. Additionally, long-term systemic immune status beyond the early postoperative period was not evaluated. Fourth, the precise molecular mechanisms by which Th7R at specific LN stations influences the peripheral blood immune landscape remain to be elucidated. Finally, although wedge resection without lymph node dissection is performed as standard clinical practice in Japan, this deviates from the NCCN guidelines, which recommend lymph node evaluation. To address these limitations, future prospective studies with larger cohorts, meticulously designed to minimize selection biases, along with mechanistic investigations, are warranted to fully validate our findings.

In conclusion, this study suggested that preserving TDLNs during surgery for early-stage lung cancer mitigates the postoperative decline in systemic Th7R, a tumor-specific Th1-like CD4^+^ T cell. Our results showed that hilar, interlobar, and peripheral LNs are the principal reservoirs for Th7R. In the setting of early-stage disease, our findings indicate a potential immunological benefit of LN-preserving strategies. Future studies are warranted to further explore how to optimally balance oncological outcomes with the preservation of systemic antitumor immunity.

## Supplementary Information

Below is the link to the electronic supplementary material.

Supplementary file1 (PDF 5872 kb)

## Data Availability

Raw data supporting the findings of this study are available from the corresponding author upon reasonable request.

## Abbreviations

ANCOVA: Analysis of covariance
DFS: Disease-free survival
ICI: Immune checkpoint inhibitor
LN: Lymph node
LND: Lymph node dissection
PBMC: Peripheral blood mononuclear cell
TDLN: Tumor-draining lymph node
TLS: Tertiary lymphoid structure
TME: Tumor microenvironment
