# Correction: Immunological impact of tumor-draining lymph node dissection on systemic Th1-like CD4^+^ T cells in patients with early-stage lung cancer

**DOI:** 10.1007/s00262-026-04490-0

**Published:** 2026-08-01

**Authors:** Atsushi Kamigaichi, Hiroshi Kagamu, Yoshihiro Miyata, Takahiro Mimae, Norifumi Tsubokawa, Koichi Hirano, Morihito Okada

**Affiliations:** 1 Department of Surgical Oncology, Hiroshima University, 1-2-3 Kasumi, Minami-ku, Hiroshima, 734-8551 Japan; 2 Division of Respiratory Medicine, Saitama Medical University International Medical Center, Saitama, Japan

**Correction to: Cancer Immunology, Immunotherapy (2026) 75:157** 10.1007/s00262-026-04357-4

In the original version of this article, the right side of Figure 2 is cut off, making several data points and labels invisible.

The Fig. 2 should have appeared as shown below.

**Fig. 2:**
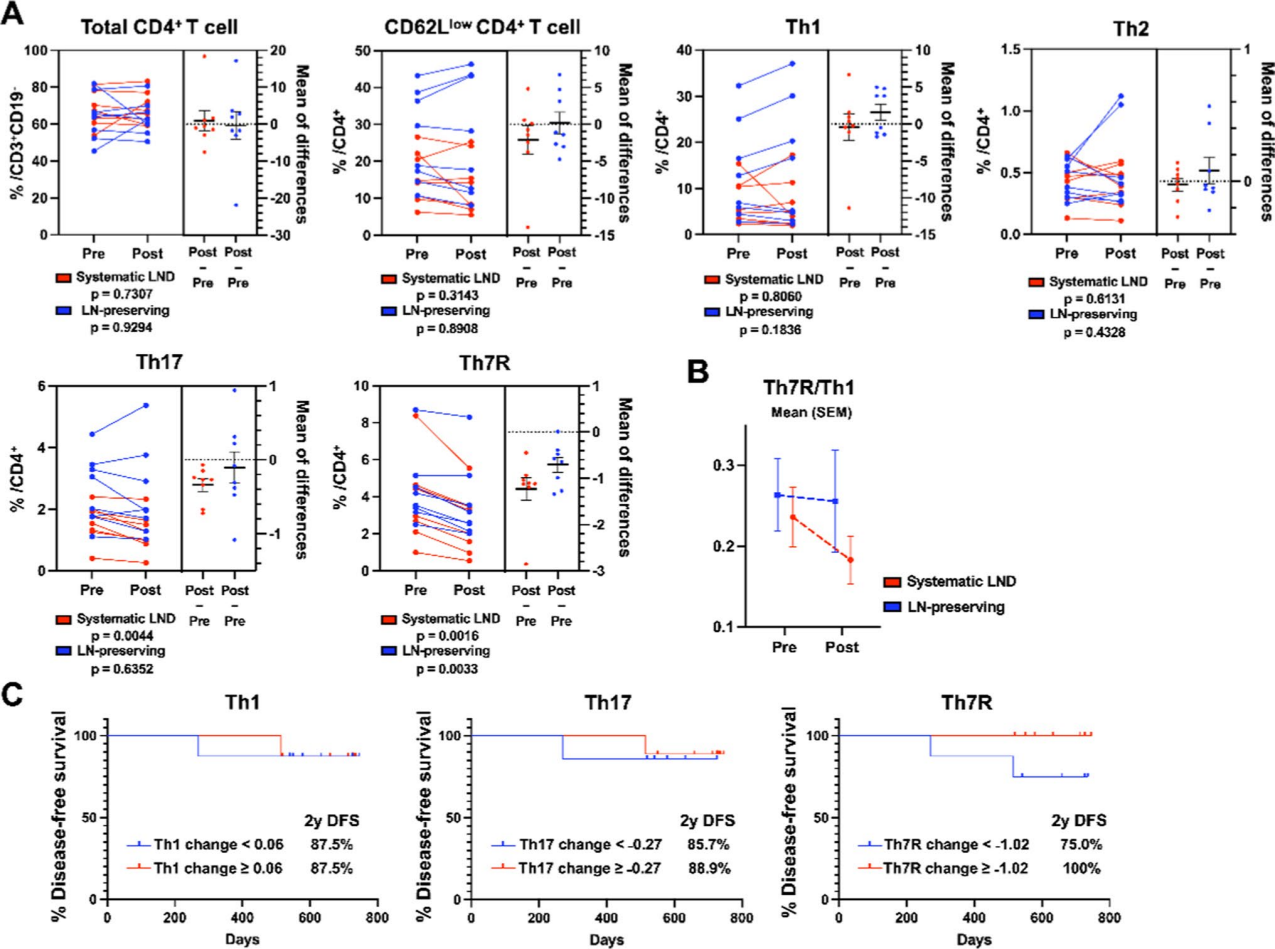
Postoperative dynamics of CD4^+^ T cell subsets in peripheral blood after lung cancer surgery **A**, Paired plots showing the preoperative and postoperative percentages of total CD4^+^ T cell, CD62L^low^CD4^+^ T cell, Th1, Th2, Th17, and Th7R in systematic lymph node dissection (LND) group (red lines) or LN-preserving group (blue lines). The right panels show the individual postoperative changes, calculated as the postoperative minus preoperative percentages. **B**, Paired analysis of the Th7R/Th1 ratio before and after surgery. Data are presented as mean (SEM). *P* values for paired comparisons were determined using the paired t test. **C**, Kaplan–Meier curves for disease-free survival (DFS) comparing patients stratified by postoperative changes in Th1, Th17, and Th7R in peripheral blood. The cutoff value was based on the median value of postoperative change (postoperative minus preoperative: Th1, 0.06%; Th17, − 0.27%; Th7R, − 1.02%). Patients were classified into the decreased (change < median) and maintained (change ≥ median) groups for each subset

The original article has been corrected.

