# Supplementary material for: Immunological impact of tumor-draining lymph node dissection on systemic Th1-like CD4+ T cells in patients with early-stage lung cancer
Source: Cancer Immunol Immunother. 2026 Apr 28;75(5):157. doi: 10.1007/s00262-026-04357-4 (PMC13125411; doi:10.1007/s00262-026-04357-4)
Supplement: Supplementary file 1 — Supplementary file1 (PDF 5872 kb) [file 262_2026_4357_MOESM1_ESM.pdf]

Supplementary Figures

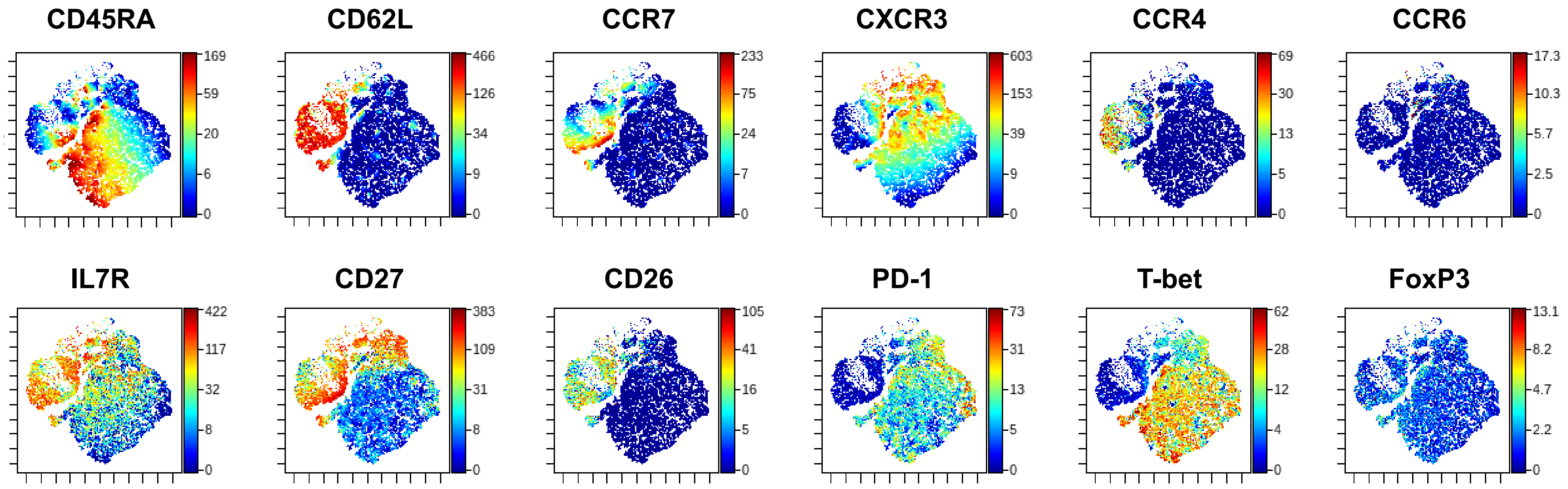

Supplementary Fig. S1 Representative viSNE figures of gated $CD8^{+}CD3^{+}$ cells from peripheral blood mononuclear cells

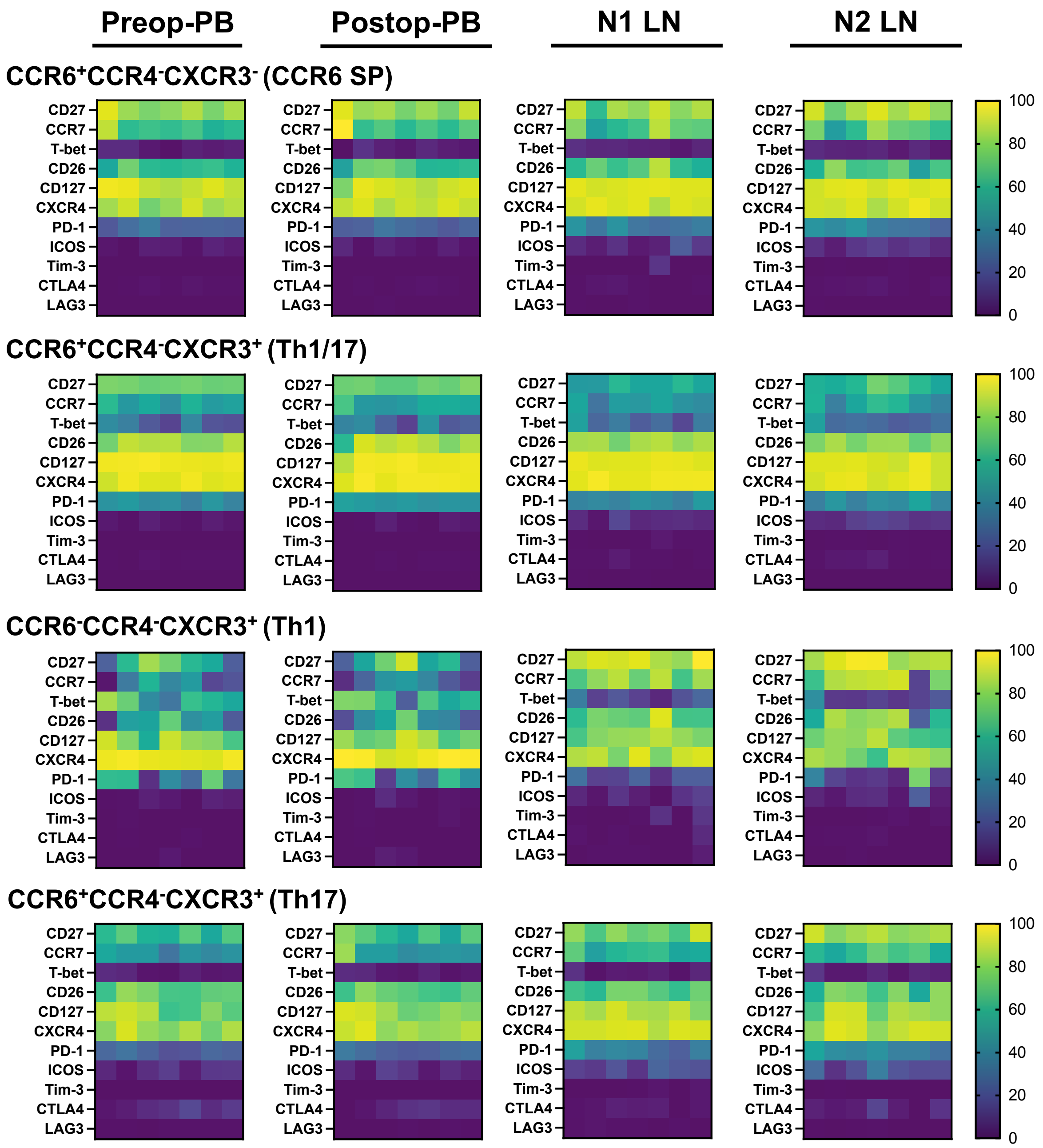

Supplementary Fig. S2 Heatmap showing expressions of key effector and inhibitory markers in $\text{CXCR3}^{-}\text{CCR4}^{-}\text{CCR6}^{+}$ (CCR6 SP), $\text{CXCR3}^{+}\text{CCR4}^{-}\text{CCR6}^{+}$ (Th1/17), $\text{CXCR3}^{+}\text{CCR4}^{-}\text{CCR6}^{-}$ (Th1), $\text{CXCR3}^{-}\text{CCR4}^{+}\text{CCR6}^{+}$ (Th17) $\text{CD62L}^{\text{low}}\text{CD4}^{+}$ T cells isolated from pre- and postoperative peripheral blood (PB), hilar/interlobar lymph nodes (N1 LNs), and mediastinal (N2 LNs) from seven patients with fully available paired samples

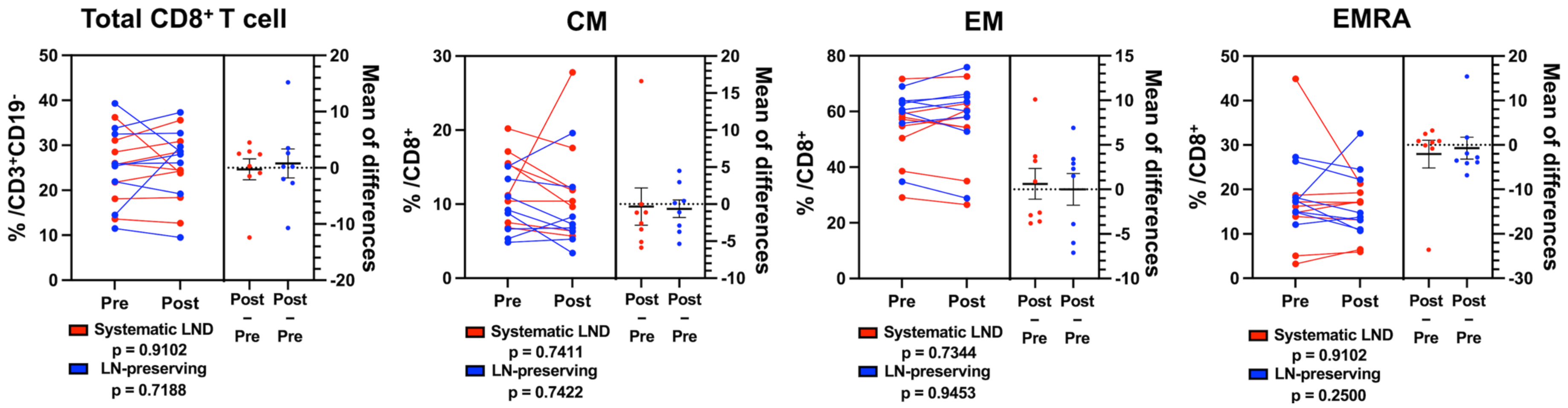

Supplementary Fig. S3 Postoperative dynamics of $CD8^+$ T cell subsets in peripheral blood after lung cancer surgery. Paired plots showing preoperative and postoperative percentages of total $CD8^+$ T cell, $CCR7^+CD45RA^-$ (central memory, CM), $CCR7^-CD45RA^-$ (effector memory, EM), and $CCR7^-CD45RA^+$ (effector memory re-expressing CD45RA, EMRA) $CD8^+$ T cells in the systematic lymph node dissection (LND) group (red lines) or lymph node (LN)-preserving group (blue lines). The right panels show individual postoperative changes, calculated as postoperative minus preoperative percentages. p-values for paired comparisons were determined using the paired t-test

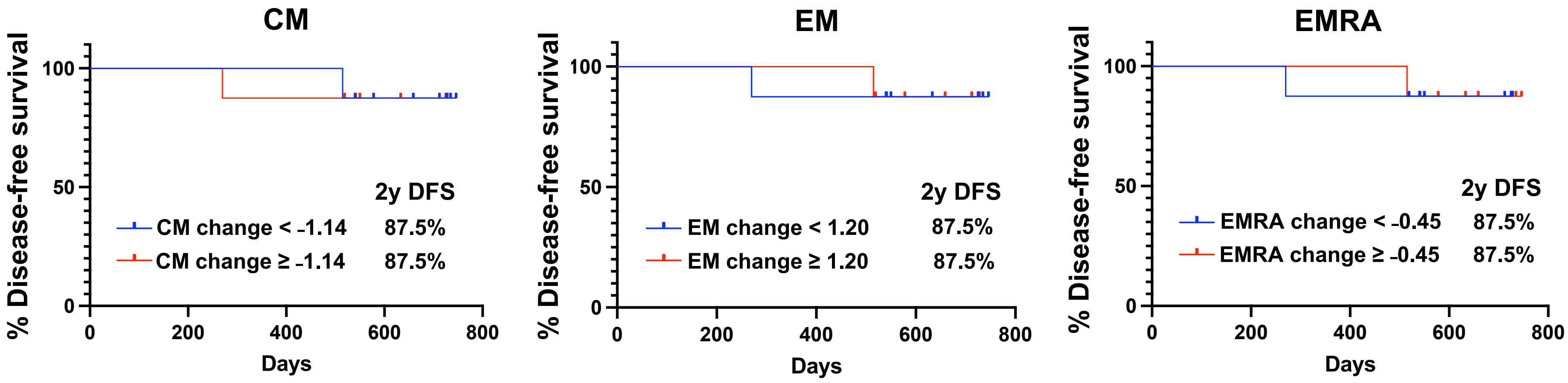

Supplementary Fig. S4 Kaplan-Meier curves for disease-free survival comparing patients stratified by postoperative changes in central memory (CM), effector memory (EM), and effector memory re-expressing CD45RA (EMRA) in peripheral blood. The cutoff value was determined based on the median postoperative change (postoperative minus preoperative: CM, -1.14%; EM, 1.20%; EMRA, -0.45%). Patients were classified into the decreased (change < median) and maintained (change ≥ median) groups for each subset

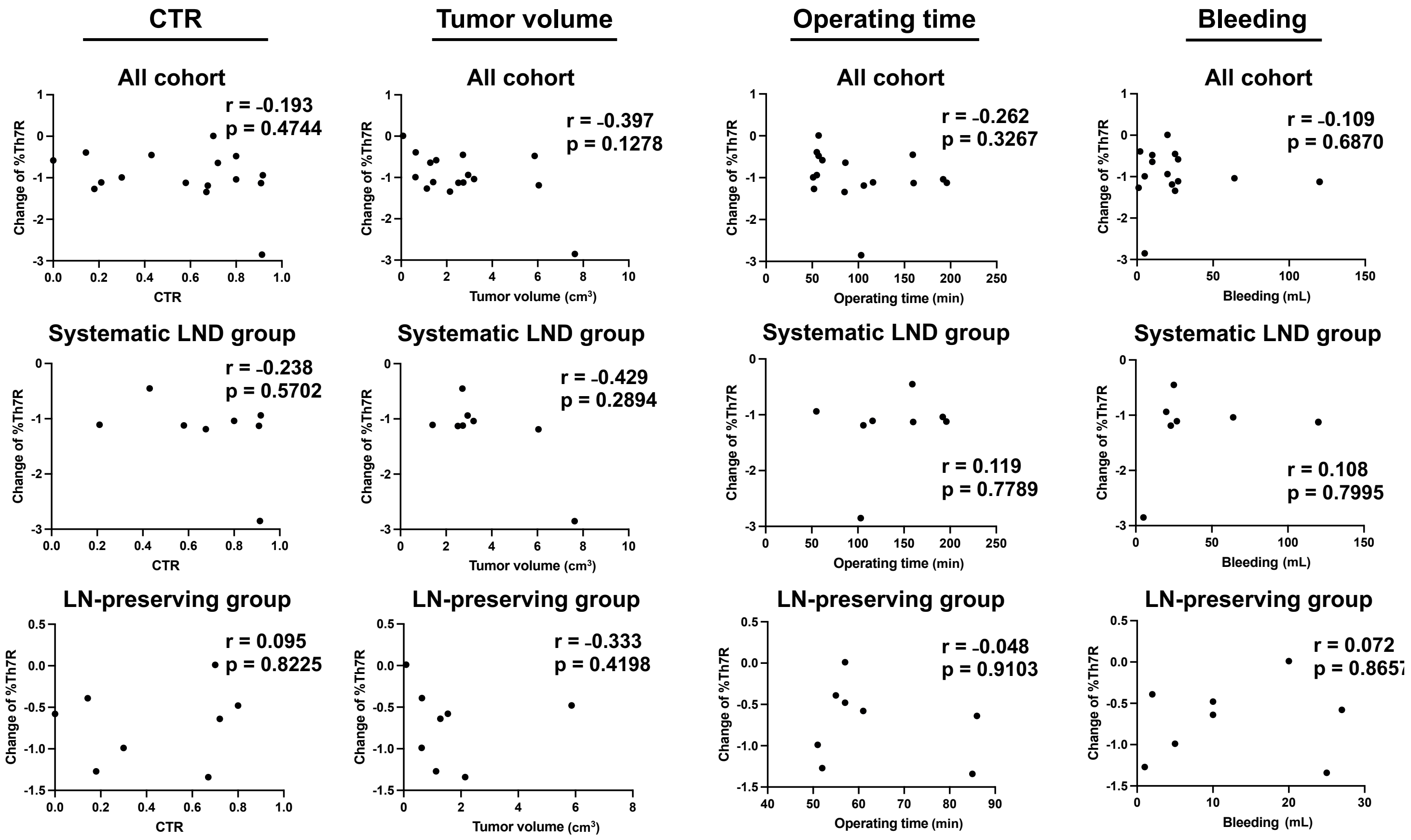

Supplementary Fig. S5 Correlation between consolidation-to-tumor ratio (CTR), tumor volume, operative time, or intraoperative blood loss and postoperative change (%) in Th7R in peripheral blood. The Spearman correlation coefficient (r) and p-value are shown for each plot
